# Old but still gold: Lithium in stabilizing the mood

**DOI:** 10.4103/0019-5545.49460

**Published:** 2009

**Authors:** Richard Balon

**Affiliations:** Department of Psychiatry and Behavioral Neurosciences, Wayne State University School of Medicine, Detroit, Michigan, USA

Many, if not all, great discoveries in psychopharmacology resulted, as Donald Klein noted,[1] “from chance observations of unexpected clinical benefits or as inadvertent outcomes of blind pharmaceutical searches.” One of such discoveries, by Cade J[2] in the late 1940's, was the calming anti-manic effect of lithium. Cade first noticed a calming effect of lithium in guinea pigs. He decided to test lithium in various mental illnesses, including mania and melancholia (interestingly, he ingested lithium himself first to ensure its safety in humans). The calming effect of lithium in mania was very robust, and Cade even speculated that mania may be caused by some lithium deficiency. His seminal discovery ultimately led to a revolutionary new approach - we not only had and have an agent to treat mania and manic-depressive illness (what we call today a bit strangely bipolar illness), but we got an agent that is useful to prevent future episodes of this illness and to stabilize fluctuations of mood. However, one should note that William Hammond first reported on the usefulness of lithium in manic states as early as in 1871, which has not been mentioned in the textbooks.[3]

While Cade[2] is rightfully given credit for the discovery of the anti-manic properties of lithium, it is not clear whether this discovery would not have remained just an interesting observation without wide-scale clinical application but for the work of other giants of the golden era of psychopharmacology. The main student and proponent of lithium use was the Danish psychiatrist Mogens Schou (1918-2005). Schou started to examine the clinical properties of lithium as a young psychiatrist during the early 1950's. Lithium remained his “life-long obsession.” In his early exploration of lithium, he designed one of the first partly open and partly randomized and placebo-controlled trials of this agent. His early research confirmed the previous finding that lithium has anti-manic properties.[4] Schou spent endless time researching all aspects of lithium's effect on the body and the brain, and published on a wide range of issues, from lithium in the heart muscle, to lithium's teratogenicity, and the effect of prolonged lithium administration on cerebral monoamine neurons in rats. His work cemented the role for lithium in the prophylaxis of manic-depressive illness, in spite of a lot of doubts and opposition (especially among the British psychiatrists). He published well over 500 articles on lithium. He remained an unfettered defender of lithium's use during the attacks on lithium, doubts about its efficacy, reports of its toxicity and possible teratogenicity. He has published excellent summaries and “anniversary,” articles on lithium treatment.[5]

In the recent era, when many new agents are being proposed (mainly by the pharmaceutical industry) as “mood stabilizers,” some may ask, “so what, there are other, easier to use mood stabilizers.” True, we have many other medications, such as valproic acid, carbamazepine, and several other anticonvulsants and antipsychotics, used as anti-manic agents or mood stabilizers. However, as Schou[5] and others pointed out, the prophylactic effect of these so called mood stabilizers remains either weak or missing. Lithium remains a unique agent, the gold standard among the so called mood stabilizers. As Bauer and Mitchner[6] stated in their review, the evidence supports a role for lithium as a first-line agent for the treatment of manic-depressive (bipolar) disorder. They proposed a “two-by-two” definition, which considers an agent a mood stabilizer if it has efficacy in treating acute manic and depressive symptoms, and prevents manic and depressive symptoms in manic-depressive disorder. After their extensive review of mood stabilizer trials,[6] they concluded that among “mood stabilizers”, only lithium fulfilled the a priori definition of a mood stabilizer (relaxing their quality criterion did not change this finding). It is important to note that they also pointed out that lithium monotherapy has been the exception rather than the rule.

The most serious outcome of manic-depressive illness is suicide. Interestingly, several reports during the last decade indicated that lithium, in addition to its mood stabilizing and other properties also possesses an anti-suicidal effect in bipolar disorder patients. Tondo and colleagues[7] in their large observational study found that lithium maintenance was associated with marked reduction in life-threatening suicidal acts - and that the numbers of these acts sharply increased after lithium discontinuation. Although treatment with other mood stabilizers may also lead to the reduction of suicide rates, it seems that lithium may have some superiority in relation to the prevention of suicide.[8]

Thus lithium is not only a unique mood stabilizer that meets various standards proposed for mood stabilizers, but also helps to prevent the most serious complication of mood disorders - suicide. Competent clinicians should continue using lithium in the treatment of acute episodes of manic-depressive illness as well as in the prophylaxis of it. Its benefits usually outweigh the side effects and other difficulties with laboratory tests for lithium levels. As long as patients are evaluated regularly for lithium's side effects on the thyroid gland and kidneys, lithium will remain as an agent that has substantial prophylactic and therapeutic effect in manic-depressive illness. As Mogens Schou[5] noted several years ago, “the clinical, methodological, economical, and human benefits of prophylactic lithium treatment are vast.” And as they remain vast, competent clinicians should not deprive their manic-depressive patients of the benefit of this still uniquely effective and fairly cheap agent.
